# Hepatotoxicity in HIV-infected children and adolescents on antiretroviral therapy

**DOI:** 10.1590/S1516-31802007000400002

**Published:** 2007-07-01

**Authors:** Ana Cecília Montes Gil, Raquel Lorenzetti, Gun Bergsten Mendes, André Moreno Morcillo, Adyléia Aparecida Dalbo Contrera Toro, Marcos Tadeu Nolasco da Silva, Maria Marluce dos Santos Vilela

**Keywords:** Aids, Pediatrics, Highly active antiretroviral therapy, Drug toxicity, Anti-bacterial agents, AIDS, Pediatria, Terapia anti-retroviral de alta atividade, Toxicidade de drogas, Agentes antibacterianos

## Abstract

**CONTEXT AND OBJECTIVE:**

Adverse drug reactions are a significant problem in patients on antiretroviral therapy (ART). We determined liver enzyme elevation frequencies in HIV-infected children and adolescents receiving ART, and their association with risk factors.

**DESIGN AND SETTING:**

Cross-sectional study, at the Pediatrics Immunodeficiency Division, University Hospital, Universidade Estadual de Campinas.

**METHODS:**

Medical records of 152 children and adolescents (54.6% male; median age 7.48 years) were analyzed, with a mean of 2.6 liver enzyme determinations per patient. Clinically, patients were classified in categories N (6), A (29), B (78) and C (39). Serum levels of aspartate aminotransferase and alanine aminotransferase were evaluated. Hepatotoxicity was scored as grade 1 (1.1-4.9 times upper limit of normality, ULN), grade 2 (5.0-9.9 times ULN), grade 3 (10.0-15.0 times ULN) and grade 4 (> 15.0 times ULN). To assess hepatotoxicity risk factors, odds ratios (OR) and adjusted odds ratios (aOR) for age, gender, TCD4^+^ cell count, viral load and medication usage were calculated.

**RESULTS:**

We observed grade 1 hepatotoxicity in 19.7 % (30/152) patients. No cases of grade 2, 3 or 4 were detected. There was a significant association between hepatotoxicity and use of sulfonamides (OR, 3.61; 95% confidence interval (CI), 1.50–8.70; aOR, 3.58; 95% CI, 1.44–8.85) and antituberculous agents (OR, 9.23; 95% CI, 1.60–53.08; aOR, 9.05; 95% CI, 1.48–55.25). No toxicity was associated with ART.

**CONCLUSIONS:**

One fifth of patients experienced mild hepatotoxicity, attributed to antituberculous agents and sulfonamides. Our results suggest that ART was well tolerated.

## RESUMO

**CONTEXTO E OBJETIVO:**

Reações adversas a drogas são um problema significativo em pacientes sob terapia antiretroviral (TARV). Determinamos a freqüência de valores elevados de enzimas hepáticas em um grupo de crianças e adolescentes infectados pelo HIV sob TARV e os fatores de risco associados.

**TIPO DE ESTUDO E LOCAL:**

Estudo transversal, realizado na Divisão de Imunodeficiência em Pediatria, Hospital das Clínicas, Universidade Estadual de Campinas.

**MÉTODOS:**

Foram analisados prontuários médicos de 152 crianças e adolescentes (54,6% masculino) infectados pelo HIV sob TARV, com dosagens de enzimas hepáticas, em média, 2,6 exames por paciente. A mediana de idade foi 7,48 anos. Clinicamente os pacientes foram classificados nas categorias N (6), A (29), B (78) e C (39). Foram avaliados os níveis séricos de aspartato aminotransferase e alanina aminotransferase. O sistema de escore da hepatotoxicidade foi: grau 1 (1,1 a 4,9 x• limite superior ao normal, *i.e.*, LSN), grau 2 (5,0 – 9,9 x LSN), grau 3 (10,0 – 15,0 x •LSN) e grau 4 (>15,0 x •LSN). Para determinar os fatores de risco de hepatotoxicidade, foram avaliados odds ratio (OR) e odds ratio ajustado (aOR) para idade, gênero, contagem de linfócitos TCD4^+^ e uso de medicações.

**RESULTADOS:**

Observamos hepatotoxicidade grau 1 em 19,7% (30/152) pacientes. Não foi detectada hepatotoxicidade grau 2, 3 ou 4. Houve uma associação significativa entre a hepatotoxicidade e uso de sulfas (OR, 3,61; IC 95%, 1,50 –8,70; OR_ajustado_, 3,58; IC 95%, 1,44 – 8,85) e agentes antituberculose (OR, 9,23; IC 95%, 1,60 – 53,08; OR_ajustado_, 9,05, IC 95%, 1,48 – 55,25), mas não estava associada com TARV.

**CONCLUSÃO:**

Um quinto dos pacientes apresentaram hepatotoxicidade leve, atribuída ao uso de agentes antituberculose e sulfas. Nossos resultados sugerem que TARV foi bem tolerada.

## INTRODUCTION

Antiretroviral therapy (ART) has had an effect on the lives of HIV-infected children and adults. Survival has improved, patients have required fewer hospitalizations, and the incidence of opportunistic infections has decreased.^1^ However, hepatotoxicity is a significant problem in patients on highly active antiretroviral therapy (HAART). In approximately 6% to 30% of treated patients, ART is associated with significantly increased serum liver enzyme levels, which may require discontinuation of treatment.^2,3^ In adults starting on ART, 14-20% will experience elevations of liver enzyme levels^4,5^ and 2-10% will need to stop receiving ART due to severe liver injury.^6^

Differential diagnoses for liver diseases in patients with HIV infection are often a difficult task. Abnormal levels of liver enzymes are common and may be caused by HIV itself, hepatitis viruses, systemic opportunistic infections, malignancies or drug-induced hepatotoxicity.^6^

The mechanisms involved in HAART-derived liver toxicity are poorly understood. Elevations in serum liver enzyme levels have been described in relation to all the major classes of antiretrovirals. The underlying mechanisms proposed have included mitochondrial toxicity relating to several nucleoside analog reverse transcriptase inhibitors (NRTIs) and hypersensitivity reactions relating to non-nucleoside reverse transcriptase inhibitors (NNRTIs).^7^

It has been estimated that 2.5 million children worldwide are living with acquired immunodeficiency syndrome (AIDS), according to data collected for the 2004 report from the UNAIDS/WHO Working Group on Global HIV/AIDS.^8^ In Brazil, 10,917 cases of AIDS were reported in children under 13 years of age from 1983 to 2004.^9^

Brazil was the first developing country to implement a large-scale universal antiretroviral distribution program. Approximately 141,000 patients are receiving free antiretroviral drugs through the public health system.^10^ However, most information relating to therapeutic toxicity comes from the settings of developed countries. There are few data on liver toxicity among the pediatric population receiving antiretroviral treatment in developing countries.

## OBJECTIVE

The aim of this study was to determine the frequencies of asymptomatic or symptomatic liver enzyme elevations in HIV-positive children and adolescents receiving ART in outpatient settings and to correlate these frequencies with risk factors.

## MATERIAL AND METHODS

This was a cross-sectional study that included all 152 HIV-infected children and adolescents who were followed up at the Pediatrics Immunodeficiency Division of the University Hospital, Universidade Estadual de Campinas, State of São Paulo, Brazil, from March 2003 to April 2004. These patients were receiving ART and had undergone measurements of serum aspartate aminotransferase (AST) and alanine aminotransferase (ALT). They were treated by specialized staff, in accordance with the guidelines established by the Brazilian Ministry of Health.^11^ The medical school's Ethics Review Board approved the study.

Data were collected from the patients’ records, including clinical and immunological categories, HIV viral load, antiretroviral drug use, antimicrobial therapy (antituberculous agents, sulfonamides, imidazoles and macrolides) and serum levels of AST and ALT.

Clinically, they were classified according to the 1994 criteria of the Centers for Disease Control and Prevention (CDC)^12^ as asymptomatic (category N), mildly symptomatic (category A), moderately symptomatic (category B) or severely symptomatic (category C). Their immunological status was defined as class 1 (no immunosuppression), 2 (moderate immunosuppression) or 3 (severe immunosuppression) on the basis of the TCD4^+^ cell count adjusted for age.

For the purpose of this report, we presumed that the antiretroviral drugs prescribed were taken; antiretroviral drug use was included in the analysis unless there was documentation that they were not taken.

### Monitoring of hepatotoxicity

During the year of reviewing the medical files, we collected data from laboratory tests on liver function and from abdominal ultrasonography. Imaging studies were performed only on patients with hepatomegaly and clinical suspicion of liver disease. The median number of repeated liver enzyme determinations was 2.6 (range: 1-5) per patient. Only the highest value of ALT and AST was considered.

ALT and AST levels were determined by using Reflotron^®^ Plus at 37° C (Roche Diagnostics, Mannheim, Germany). Titers were expressed in U/l.

The medical records were reviewed to exclude other potential causes of liver disease, such as Epstein-Barr virus (EBV), cytomegalovirus (CMV) and hepatitis A, B and C. Liver biopsy was performed on only one patient.

Hepatic toxic effects were graded according to the toxicity tables of the AIDS Division of the Pediatrics AIDS Clinical Trials Group.^13^ Hepatotoxicity was considered to be present when ALT and AST levels rose above the upper limits of normality (ULN). Normality was considered to be the values defined by Fischbach and Zawta in 1992.^14^ The scoring system was the following: grade 1 (1.1 - 4.9 x ULN), grade 2 (5.0 - 9.9 x ULN), grade 3 (10.0 - 15.0 x ULN) and grade 4 (> 15.0 x ULN).

In the group presenting hepatotoxicity, the clinical data and ALT and AST levels were reviewed from the beginning of the clinical follow-up at our service, in order to rule out previous liver disease.

Peripheral T lymphocytes and their subpopulations were quantified by flow cytometry in FACS Count^®^ equipment (Beckton-Dickinson, Franklin Lakes, NJ, USA). The results were expressed as the total number of cells/mm^3^.

The NucliSens^®^ assay (Organon-Teknika, Durham, NC, USA) was used to determine quantitative plasma HIV-1 RNA levels, with a lower limit of quantification of 80 RNA copies/ml. The assays were performed in the certified AIDS laboratory at our institution, in accordance with the standards recommended by the Brazilian Health Ministry.^15^

### Data analysis

All data collected were organized into a database using Excel software (Microsoft^®^ 2000, Microsoft, Redmond, USA). The statistical analysis was performed by means of the Statistical Package for the Social Sciences (SPSS) software, version 11.0 (SPSS, Chicago, USA). Associations between dependent and independent variables were initially assessed using univariate analysis. Odds ratios (OR) were calculated, and the chi-squared or Fisher exact test was used. Variables that showed significant associations with hepatotoxicity risk were then assessed by multivariate analysis, with adjusted odds ratios (aOR), using unconditional logistic regression and the forward stepwise method, and probability levels of 0.05 and 0.10, respectively. We investigated associations between the occurrence of liver abnormalities in laboratory tests and the clinical variables evaluated. The variables included in the basic model were age, gender, immunological category, last viral load and antimicrobial therapy. The use of antituberculous agents, sulfonamides, imidazoles and macrolides was analyzed separately. We also did analysis on the commonly coprescribed antiretrovirals (zidovudine, lamivudine, didanosine, stavudine, efavirenz, nevirapine, nelfinavir, ritonavir and lopinavir), to improve the power of the logistic model to separate associations.

## RESULTS

Among the 152 children and adolescents receiving ART, 54.6% were boys. Their ages ranged from 0.6 to 18.2 years (median: 7.48). The mean TCD4^+^ lymphocyte count was 690.8 cells/mm^3^. The data on the patients’ characteristics collected cross-sectionally are displayed in Table 1.

**Table 1.** Hepatotoxicity in HIV-infected children and adolescents on antiretroviral therapy, in relation to gender, viral load and classification. Data were collected from March 2003 to April 2004 in a university hospital

	Hepatotoxicity		
	Yes	No	OR (95% CI)
**Gender**			
Male	13	69	1.70 (0.76-3.81)
Female	17	53	
**Viral load (copies/ml)**			
> 100,000	3	2	6.66 (1.06-41.85)
< 100,000	27	120	
**Immunological category**			
1	5	19	1.00
2	8	61	0.49 (0.14-1.70)
3	17	42	1.53 (0.49-4.78)
**Clinical category**			
N + A	5	30	1.00
B	13	65	1.19 (0.39-3.67)
C	12	27	2.66 (0.83-8.54)

*OR = odds ratio; CI = confidence interval; N = asymptomatic; A = mildly symptomatic; B = moderately symptomatic; C = severely symptomatic.*

While 148 children had been infected with HIV vertically, four had been infected through blood transfusion. Using the CDC guidelines^12^ the clinical classification of the pediatric HIV infection was that there were six patients in category N, 29 in A, 78 in B and 39 in C.

**Figure 1.**
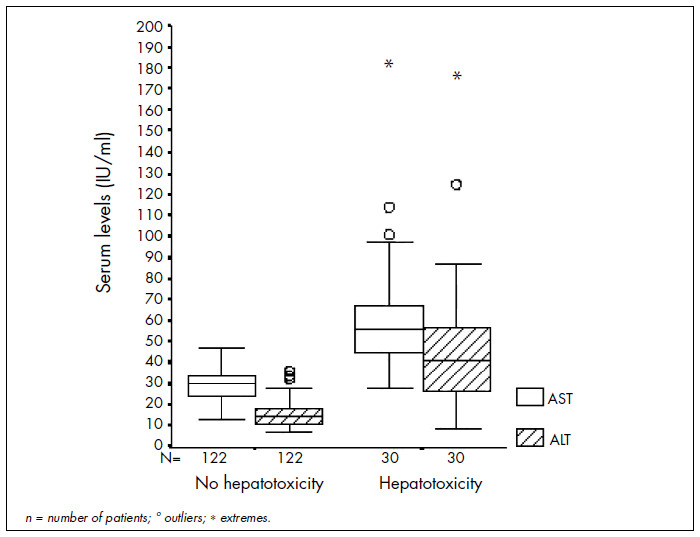
Box-plot showing distribution of aspartate aminotransferase (AST) and alanine aminotransferase (ALT) serum levels according to hepatotoxicity categories in HIV-positive children and adolescents followed up in a university hospital.

Thirty patients (19.7%) had elevations in transaminase levels: 20 of them in AST and 14 in ALT. The hepatotoxicity score was limited to grade 1. Overall, the mean ALT level was 22 U/l (standard deviation, SD = 21.1 U/l) and the mean AST level was 36.5 U/l (SD = 19.7 U/l). These results are summarized in Figure 1.

Twelve out of the 30 patients presenting liver enzyme abnormalities underwent abdominal ultrasonography. Among these, seven (58.3%) had a diagnosis of hepatosplenomegaly with hepatic steatosis. Abdominal pain was reported in three patients.

One patient had hepatitis C and another patient had histoplasmosis, which probably were the causes of their transient elevation of transaminase levels.

With regard to ART, the 152 study subjects were receiving combinations of nucleoside analogue reverse transcriptase inhibitors (NRTIs), non-nucleoside reverse transcriptase inhibitors (NNRTIs) or protease inhibitors, in accordance with national guidelines^11^ (Table 2). Assessment of the antiretroviral regimens indicated that 15.8% of the patients (24/152) were on double therapy, 75.7% (115/152) on triple therapy and 8.6% (13/152) were on four-drug regimens.

**Table 2.** Hepatotoxicity in HIV-infected children and adolescents followed up in a university hospital, in relation to antiretroviral therapy

Drug	Hepatotoxicity		
	Yes	No	OR (95% CI)
**Stavudine**			
Yes	10	25	1.93 (0.80-4.66)
No	20	97	
**Lamivudine**			
Yes	12	56	0.78 (0.34-1.77)
No	18	66	
**Zidovudine**			
Yes	20	97	0.51 (0.21-1.23)
No	10	25	
**Didanosine**			
Yes	18	66	1.27 (0.56-2.86)
No	12	56	
**Nevirapine**			
Yes	1	1	4,17 (0.25-68.69)
No	29	121	
**Efavirenz**			
Yes	5	12	1,83 (0.59-5.67)
No	25	110	
**Lopinavir/ritonavir**			
Yes	4	7	2.52 (0.68-9.27)
No	26	115	
**Ritonavir**			
Yes	4	18	0.88 (0.27-2.85)
No	26	104	
**Nelfinavir**			
Yes	17	71	0.93 (0.41-2.10)
No	13	51	

*OR = odds ratio; CI = confidence interval.*

In addition, 35 patients were on prophylaxis or therapy for opportunistic infections. Among these, 27 received trimethoprim/sulfamethoxazole (TMP-SMX), eight azithromycine, two ketoconazole, three fluconazole, six isoniazid, three rifampicin, two ethambutol, four sulfadiazine and pyrimethamine (Table 3).

**Table 3.** Multiple logistic regression for hepatotoxicity risk factors in HIV-positive children and adolescents followed up in a university hospital

	Hepatotoxicity			
	Yes	No	OR (95% CI)	aOR (95% CI)
**Viral load (copies/ml)**				
> 100,000	3	2	6.66 (1.06-41.85)	1.78 (0.22-14.11)
< 100,000	27	120		
**Drugs**				
**Antituberculous**				
Yes	4	2	9.23 (1.60-53.08)	9.05 (1.48-55.25)
No	26	120		
**Sulfonamides**				
Yes	12	9	3.61 (1.50-8.70)	3.58 (1.44-8.85)
No	18	103		
**Antifungal azoles**				
Yes	1	4	1.01 (0.10-9.44)	ND
No	29	118		
**Macrolides**				
Yes	2	7	1.17 (0.23-5.95)	ND
No	28	115		

*OR = odds ratio; CI = confidence interval; aOR = adjusted odds ratio; ND = not done (non-significant odds ratio).*

The multiple logistic regression model revealed that hepatotoxicity was associated only with the use of sulfonamides (aOR: 3.58; 95% confidence interval, CI: 1.44 – 8.85) and antituberculous agents (aOR: 9.05; 95% CI: 1.48 – 55.25) (Table 3). Coadministration of antituberculous agents for four patients out of the 30 presenting hepatotoxicity (13.3%), consisting of an association of isoniazid and rifampicin (two cases) or isoniazid alone as a prophylactic (two cases), could have contributed to the transitory increase in liver enzymes.

## DISCUSSION

Mild liver toxicity was observed in 19.7% of the HIV-infected children and adolescents on ART. This result suggests that this population tolerated ART well, probably because of an absence of risk factors that would contribute towards the development of severe liver injury in adults such as hepatitis C virus and/or hepatitis B virus coinfection, older age, high alcohol intake and use of illicit drugs.^2,16^

Grade 1 hepatotoxicity, as assessed by AST levels, was found in three patients (8%) out of a group of 36 HIV-1 infected children who were treated with four or five antiretroviral agents.^17^ Another recent study^18^ showed that 16% of 43 children on ART developed hepatotoxicity with elevated liver enzyme levels at least five times the baselines values.

Hepatotoxicity ranges in severity from the absence of symptoms to liver insufficiency, and the outcomes range from spontaneous resolution to liver failure and death. Low-grade hepatotoxicity is a warning for physicians caring for patients on ART.^16^

Liver enzyme elevations are common in HIV-infected patients, especially those treated with HAART. Despite such reports, analysis of the events surrounding liver enzyme elevations is limited, because HIV-infected patients present several risk factors for biochemical abnormalities, and a precise etiology is rarely defined clearly.^19^

Other than HAART-derived hepatotoxicity, some liver diseases are often associated with HIV infection and should also be ruled out.^19^ Our results showed no significant relationship between grade 1 hepatotoxicity and higher viral load (≥ 100,000 copies/ml), while coexistence with hepatitis C or histoplasmosis infection was documented in two isolated patients.

Use of sulfonamides and coadministration of antituberculous agents were significantly associated with grade 1 hepatotoxicity. Differently to an earlier report,^18^ the hepatotoxicity observed in our study was not related to ART or to clinically and immunologically compromised conditions.

HAART is a combination therapy of several antiretroviral drugs and thus can cause hepatotoxic drug interactions by itself. In particular, NNRTIs and protease inhibitors interact with cytochrome P450 and thus mutually alter their serum half-lives.^19^ A combination regimen of ritonavir, zidovudine, and lamivudine was found to be generally safe and produced sustained viral suppression in more than one third of infants who started therapy before two years of age.^20^

The finding that 15.8% of our patients continued to receive dual-ART in 2004 can probably be attributed to the fact that these patients continued to have low viral loads and adequate immune function. In accordance with the guidelines for prescribing three antiretroviral drugs initially, preferably including a protease inhibitor or an NNRTI, most children in this study were receiving triple therapy. The survival rates have increased in association with triple antiretroviral use.^21^ As children and adolescents on ART go through hormonal changes and growth spurts associated with puberty, the late complications and toxicity of chronic exposure to HAART also need to be carefully monitored and evaluated.

Many common antibiotics, antifungals and antivirals prescribed for HIV-infected patients are independently associated with hepatotoxicity. Drugs commonly used for HIV-infected patients can induce cholestatic (TMP-SMX) or hepatocellular (isoniazid) patterns.^16^

In addition to predisposing towards tuberculosis, AIDS may predispose towards the development of drug-induced hepatotoxicity secondary to the use of antituberculous agents.^22^ One analysis indicated a fourfold increased risk of drug-associated hepatitis in patients treated simultaneously for HIV and tuberculosis.^23^ Treatment of tuberculosis constitutes a particularly difficult problem among patients on HAART, because tuberculostatic drugs themselves carry a considerable risk of inducing toxic liver damage.^24^ A meta-analysis^25^ has shown that rifampicin and isoniazid, when coadministered, might lead to synergistic hepatotoxicity. The mean incidences of drug-related toxic hepatitis were found to be 1.6% (isoniazid), 1.1% (rifampicin) and 2.6% (isoniazid + rifampicin). In our study, as in others,^16,22,23,24^ the hepatotoxicity was mainly related to isoniazid alone or in association with rifampicin.

We have described clinical and laboratory disorders associated with a wide variety of treatment combinations. In this study, patients were not randomly allocated to treatment. Allocation of treatment could have been biased, since patients with more advanced disease could have been given three classes of antiretrovirals.

A longer-term study might give a more precise understanding of the cumulative toxic effects. However, such a study would also be more complex in view of the treatment modifications and intercurrent illnesses expected during an extended follow-up.

## CONCLUSION

In conclusion, one fifth of all HIV-infected children and adolescents in this study had a high concentration of transaminases, characterized by hepatotoxicity grade 1. In addition, the risk factors for hepatotoxicity associated with such patients were concurrent use of sulfonamides and coadministration of antituberculous agents.
